# Supplementary material for: The discovery of an in situ Neanderthal remain in the Bawa Yawan Rockshelter, West-Central Zagros Mountains, Kermanshah
Source: PLoS One. 2021 Aug 26;16(8):e0253708. doi: 10.1371/journal.pone.0253708 (PMC8389444; doi:10.1371/journal.pone.0253708)
Supplement: Attachment — Submitted filename: Review of the paper.docx [file pone.0253708.s001.docx]

Review of the paper

Main Manuscript for The discovery of an in situ Neanderthal remain in the Bawa Yawan Rockshelter, West-Central Zagros Mountains, Kermanshah

By Saman Heydari-Guran et al.

The ms reports the discovery of a deciduous Neanderthal tooth from a rockshelter in the Zagros Mountains, Iran. It describes the new site, discussing the stratigraphic sequence analysed so far and provides a chronological framework of the finding through a series of C14 dates. The ms provides interesting new information on an area of Eurasia poorly known in term of Paleolithic occupation. The ms is well structured is some sections, but not in others, and it requires some reworking.

Title:

There is an obvious mistake in the outline of the title, and I suggest modifying it as follows:

The discovery of an in situ Neanderthal tooth in the Bawa Yawan Rockshelter, West-Central Zagros Mountains, Kermanshah, Iran.

Outline.

The usual subdivision in “materials and methods“ does not seem appropriate for this kind of ms.

I suggest modifying it as follows:

1. Introduction
2. Study area

2.1 Bawa Yawan Rockshelter and stratigraphy

The paragraph at line 249 – 251:

*At a depth of around 2.50 m from the surface in the upper part of GH5, where the density of MP cultural materials gradually reduced, an in situ hominin tooth (hereafter called BY1) was recovered in association with fauna and Zagros Mousterian lithic artefacts.*

should go at the end of the description of the GH 5 horizon at line 193.

2.2 Cultural materials and artefact technology and typology

The materials and methods section should be

3. Analytical methods

and include

3.1 µCT images of BY1 – this section should rather be titled “Morphological and metrical analysis of BY1.

lines 132, 133 and 134 (up to ‘according to Moorrees et al (35, 36).’ ) should be moved before line 124

3.2 Radiocarbon Dating

4. Results

4.1 BY1 Neanderthal tooth (starting from line 251)

4.2 Chronology

5. Discussion

Other comments:

Line 124- 125. Details on where the Nikon system is located should be provided.

Line 132 – 133. The ASUDAS is relative to permanent teeth. Please add a sentence to explain that ASUDAS has been adjusted.

Line 142. Reference 38 is wrong. Please check. Should be 37 ?

Line 154 and line 157 check references 38, 39 and 40.

Line 256. Reference 43 is repeated. See ref. 34.

Line 263 264: move to the description line 253.

In table 1 the 5 NEA specimens lower dc are indicated as ‘this work’. Specimens should be spelled out.

The Discussion is poorly written, with a number of grammar mistakes, and must be revised for content and for flow.

Ref. 37 in incomplete

Figure 4 Part B is not described in the caption.

Figure 5 is missing.

Section 1 and Section 2 of the Supplementary information are indeed an interesting piece of information and brings new data on this poorly known area of Palaeolithic occupation. Given that PLOS One has no page limits, the authors should consider moving this section into the main text.
