# Supplementary material for: The discovery of an in situ Neanderthal remain in the Bawa Yawan Rockshelter, West-Central Zagros Mountains, Kermanshah
Source: PLoS One. 2021 Aug 26;16(8):e0253708. doi: 10.1371/journal.pone.0253708 (PMC8389444; doi:10.1371/journal.pone.0253708)
Supplement: Attachment — Submitted filename: Response to Review of the paper 2.docx [file pone.0253708.s002.docx]

Main Manuscript for The discovery of an in situ Neanderthal remain in the Bawa Yawan Rockshelter, A

Journal Requirements:

Please review your reference list to ensure that it is complete and correct. If you have cited papers that have been retracted, please include the rationale for doing so in the manuscript text, or remove these references and replace them with relevant current references. Any changes to the reference list should be mentioned in the rebuttal letter that accompanies your revised manuscript. If you need to cite a retracted article, indicate the article’s retracted status in the References list and also include a citation and full reference for the retraction notice.

When submitting your revision, we need you to address these additional requirements.

6. We note that Figures 1 and 3 in your submission contain map images which may be copyrighted. All PLOS content is published under the Creative Commons Attribution License (CC BY 4.0), which means that the manuscript, images, and Supporting Information files will be freely available online, and any third party is permitted to access, download, copy, distribute, and use these materials in any way, even commercially, with proper attribution. For these reasons, we cannot publish previously copyrighted maps or satellite images created using proprietary data, such as Google software (Google Maps, Street View, and Earth). For more information, see our copyright guidelines: <http://journals.plos.org/plosone/s/licenses-and-copyright>.

 We require you to either (1) present written permission from the copyright holder to publish these figures specifically under the CC BY 4.0 license, or (2) remove the figures from your submission:

6.1.    You may seek permission from the original copyright holder of Figures 1 and 3 to publish the content specifically under the CC BY 4.0 license.

We recommend that you contact the original copyright holder with the Content Permission Form (<http://journals.plos.org/plosone/s/file?id=7c09/content-permission-form.pdf>) and the following text:

“I request permission for the open-access journal PLOS ONE to publish XXX under the Creative Commons Attribution License (CCAL) CC BY 4.0 (<http://creativecommons.org/licenses/by/4.0/>). Please be aware that this license allows unrestricted use and distribution, even commercially, by third parties. Please reply and provide explicit written permission to publish XXX under a CC BY license and complete the attached form.”

Please upload the completed Content Permission Form or other proof of granted permissions as an "Other" file with your submission.

In the figure caption of the copyrighted figure, please include the following text: “Reprinted from [ref] under a CC BY license, with permission from [name of publisher], original copyright [original copyright year].”

?????????????????????/

6.2.    If you are unable to obtain permission from the original copyright holder to publish these figures under the CC BY 4.0 license or if the copyright holder’s requirements are incompatible with the CC BY 4.0 license, please either i) remove the figure or ii) supply a replacement figure that complies with the CC BY 4.0 license. Please check copyright information on all replacement figures and update the figure caption with source information. If applicable, please specify in the figure caption text when a figure is similar but not identical to the original image and is therefore for illustrative purposes only.

The following resources for replacing copyrighted map figures may be helpful:

USGS National Map Viewer (public domain): <http://viewer.nationalmap.gov/viewer/>

The Gateway to Astronaut Photography of Earth (public domain): <http://eol.jsc.nasa.gov/sseop/clickmap/>

Maps at the CIA (public domain): <https://www.cia.gov/library/publications/the-world-factbook/index.html> and <https://www.cia.gov/library/publications/cia-maps-publications/index.html>

NASA Earth Observatory (public domain): <http://earthobservatory.nasa.gov/>

Landsat: <http://landsat.visibleearth.nasa.gov/>

USGS EROS (Earth Resources Observatory and Science (EROS) Center) (public domain): [http://eros.usgs.gov/#](http://eros.usgs.gov/)

Natural Earth (public domain): <http://www.naturalearthdata.com/>

?????????????????????/

7. Please upload a copy of Figure 5, to which you refer in your text on page 9. If the figure is no longer to be included as part of the submission please remove all reference to it within the text.

**Fig. 5 is included.**

8. Please include captions for your Supporting Information files at the end of your manuscript, and update any in-text citations to match accordingly. Please see our Supporting Information guidelines for more information: http://journals.plos.org/plosone/s/supporting-information.

?????????????????????/

[Note: HTML markup is below. Please do not edit.]

Reviewers' comments:

Reviewer's Responses to Questions

**Comments to the Author**

1. Is the manuscript technically sound, and do the data support the conclusions?

The manuscript must describe a technically sound piece of scientific research with data that supports the conclusions. Experiments must have been conducted rigorously, with appropriate controls, replication, and sample sizes. The conclusions must be drawn appropriately based on the data presented.

Reviewer #1: Yes

Reviewer #2: Yes

2. Has the statistical analysis been performed appropriately and rigorously?

Reviewer #1: N/A

Reviewer #2: Yes

3. Have the authors made all data underlying the findings in their manuscript fully available?

The [PLOS Data policy](http://www.plosone.org/static/policies.action#sharing) requires authors to make all data underlying the findings described in their manuscript fully available without restriction, with rare exception (please refer to the Data Availability Statement in the manuscript PDF file). The data should be provided as part of the manuscript or its supporting information, or deposited to a public repository. For example, in addition to summary statistics, the data points behind means, medians and variance measures should be available. If there are restrictions on publicly sharing data—e.g. participant privacy or use of data from a third party—those must be specified.

Reviewer #1: Yes

Reviewer #2: Yes

4. Is the manuscript presented in an intelligible fashion and written in standard English?

PLOS ONE does not copyedit accepted manuscripts, so the language in submitted articles must be clear, correct, and unambiguous. Any typographical or grammatical errors should be corrected at revision, so please note any specific errors here.

Reviewer #1: Yes

Reviewer #2: Yes

5. Review Comments to the Author

Please use the space provided to explain your answers to the questions above. You may also include additional comments for the author, including concerns about dual publication, research ethics, or publication ethics. (Please upload your review as an attachment if it exceeds 20,000 characters)

Reviewer #1: The ms provides interesting new information on an area of Eurasia poorly known in term of Paleolithic occupation. The ms is well structured is some sections, but not in others, and it requires some reworking.

Reviewer #2: The article entitled "The discovery of an in situ Neanderthal remain in the Bawa Yawan Rockshelter, West-Central Zagros Mountains, Kermanshah" by Heydari-Guran and colleagues, reports the discovery of a deciduous Neanderthal tooth from the Zagros Mountains region in Iran. The specimen (BY 1) has been dated to between ~43,600 and ~41,500 years ago, thus providing the most recent date for the presence of Neanderthals in the region. From this point of view, despite its fragmentary nature, BY 1 offers important clues for understanding the dynamics of the transition between the Middle to Upper Palaeolithic transition and the extinction of the Neanderthals in the Middle East and also, more generally, throughout the Central-Western regions of Eurasia. The anatomical description of the tooth provided by the authors is rigorous and its attribution to Homo neanderthalensis appears fully justified on the basis of its morphometric features and the stratigraphic position within the MP level labeled GH4. In the article, the authors provide a convincing reconstruction of the demographic and ecological events of expansion and extinction of human populations in the Middle East during the transition between MP and UP. For these reasons, the article is of definite scientific interest and I am pleased to recommend its publication pending some minor revisions.

My main perplexity concerns the chronological horizon (~40,000-37,000 cal BP) in which the authors place the disappearance of Neanderthals in Europe. The definition of this horizon, and in particular of the most recent date of 37,000, is particularly important in an article such as this one that focuses on the timing of Neanderthal extinction in comparison between different geographical areas and should, in my opinion, be better justified (please see my specific comment below for more details).

Revisions by lines:

Line 17: word misspelled

**Done!**

In the abstract and later in the text in lines 20-21, 39, 363 the authors report the chronological horizon ~ 40,000-37,000 cal BP as the most recent date for the disappearance of Neanderthals in Europe. This statemet is justified on the basis of articles reported in bibliography as 1-4 and 22 with particular reference to articles by Higham and colleagues (2014, ref.2), Talamo and colleagues (2020, ref. 3), and Zilhão and colleagues (2017, ref.4). However, since the recent dating of the Neanderthal fossil remains from Spy to more than 40 thousand years ago (Devièse and colleagues, 2021 doi.org/10.1073/pnas.2022466118) there is to my knowledge no Neanderthal human fossil remains that have returned a firm date less than 40 thousand years BP. The most recent dates available are based solely on archaeological levels of the late Mousterian or of the so-called transitional industries. The dates reported by Zilhão and colleagues (2017) for the Spanish site were calibrated with IntCal13 (Reimeretal., 2013) inCalib7.0.4 (Stuiver and Reimer, 1993) and are somewhat controversial. Regarding the dates reported in Talamo and colleagues (2020), it seems to me, but Talamo may contradict me, that all the dates most recent than 40,000-year ago (and in particular those at 37,000 years BP) refer to levels with transitional industries (Châtelperronian) and not to levels with final Mousterian.
Regardless of the views of the authors of this reviewed article, some of whom are among the leading proponents of the association between Chatelperronian industries and Neanderthals, the issue remains controversial and there is no unanimous agreement among scholars about such association. Thus, the 37,000 year old date for the extinction of Neanderthals in Europe only stands on the assumption that the Chatelperronian tools (of that period) were made by Neanderthals, as there is no fossil or archaeological evidence for the final Mousterian more recent than 40,000 years ago. So, about the date of 37,000 years BP as the LAD for the Neanderthals I think that the autors should specify more clearly in text to which industries they are referring to and of the evidences of their possible association with Neanderthals. This justification would not be necessary if the focus the authors themselves have given to the article was not specifically on the dynamics of Neanderhal extinction and the comparison between different geographical areas including Europe.


lines 191 and 212: only genus and species names should be italicised, not the names of taxonomic families.
**done.**

line 192: Please add "excavation" after "seasons"
**done.**

Lines 340 and 341: on the basis of the dates and descriptions provide for levels GH3-Gh5 the most recent date of 39 thousand years for the Mousterian in the region seems to be not "probable" (as reported in line 340) but only "possible".
**done.**

The article is an important contribution to the knowledge of the Neanderthals in the East and about the population and demographic dynamics and the tempo and mode of their extinction. I thank the editor for giving me the opportunity to review this fine article. I have no corrections to point to for the SI. I am not an anonymous referee.

6. PLOS authors have the option to publish the peer review history of their article ([what does this mean?](https://journals.plos.org/plosone/s/editorial-and-peer-review-process#loc-peer-review-history)). If published, this will include your full peer review and any attached files.


If you choose “no”, your identity will remain anonymous but your review may still be made public.


**Do you want your identity to be public for this peer review?** For information about this choice, including consent withdrawal, please see our [Privacy Policy](https://www.plos.org/privacy-policy).

Reviewer #1: No

Reviewer #2: **Yes:** Fabio Di Vincenzo

[NOTE: If reviewer comments were submitted as an attachment file, they will be attached to this email and accessible via the submission site. Please log into your account, locate the manuscript record, and check for the action link "View Attachments". If this link does not appear, there are no attachment files.]

While revising your submission, please upload your figure files to the Preflight Analysis and Conversion Engine (PACE) digital diagnostic tool, <https://pacev2.apexcovantage.com/>. PACE helps ensure that figures meet PLOS requirements. To use PACE, you must first register as a user. Registration is free. Then, login and navigate to the UPLOAD tab, where you will find detailed instructions on how to use the tool. If you encounter any issues or have any questions when using PACE, please email PLOS at [figures@plos.org](mailto:figures@plos.org). Please note that Supporting Information files do not need this step.
